# Improving Outcomes with Sequential Fixation Using Long-Threaded Screws for Valgus-Impacted Femoral Neck Fractures

**DOI:** 10.3390/medicina61010040

**Published:** 2024-12-30

**Authors:** Jeong-Hyun Koh, Seungyeob Sakong, Won-Tae Cho, Sumin Lim, Hyung Keun Song

**Affiliations:** Department of Orthopedic Surgery, Ajou University School of Medicine, Suwon 16499, Republic of Korea; osboy513@gmail.com (J.-H.K.); sgsy4040@gmail.com (S.S.); ccarius85@gmail.com (W.-T.C.); khoo1003@gmail.com (S.L.)

**Keywords:** femoral neck fractures, valgus impacted, fully threaded screws

## Abstract

*Background and Objectives:* Valgus-impacted femoral neck fractures (OTA 31B1.1 and 31B1.2) are considered stable fractures with favorable outcomes compared to displaced fractures. However, complications such as femoral neck shortening, screw sliding, and suboptimal recovery can occur, particularly in severe deformities. This study evaluated the outcomes of a sequential fixation technique using short-threaded screws followed by long-threaded screws. *Materials and Methods:* This prospective study included 135 patients aged 60 years or older with valgus-impacted femoral neck fractures (OTA 31B1.1 and 31B1.2) treated between March 2017 and February 2021. Patients were divided into two groups: those treated solely with short-threaded screws (the control group) and those treated using a sequential fixation technique involving initial compression with short-threaded screws followed by stabilization with long-threaded screws. Exclusion criteria included follow-up < 12 months, pathological fractures, high-energy trauma, or periprosthetic fractures. Clinical outcomes, including the Harris Hip Score (HHS), and radiological parameters, such as screw sliding distance (SDS) and fixation failure, were analyzed. Multivariate regression identified predictors of outcomes to assess the effectiveness of the sequential fixation technique. *Results:* The mean follow-up was 38.3 months. Multivariate regression revealed that posterior tilt > 15° (β = 2.944, *p* < 0.001) and the use of long-threaded screws (β = −1.906, *p* < 0.001) were significant predictors of reduced SDS. Posterior tilt > 15° (OR 15.085, *p* = 0.002), valgus tilt > 15° (OR 28.616, *p* = 0.002), and bone mineral density (OR 0.285, *p* = 0.005) were predictors of fixation failure, while long-threaded screws significantly reduced fixation failure risk (OR 0.062, *p* = 0.005). *Conclusions:* The sequential use of short-threaded screws for compression, followed by long-threaded screws for stabilization, effectively reduced screw sliding and fixation failure while improving functional and radiological outcomes. This technique shows promise as an effective treatment for valgus-impacted femoral neck fractures.

## 1. Introduction

Valgus-impacted femoral neck fractures, classified as OTA 31B1.1 and B1.2, are generally considered stable fractures with better outcomes than displaced fractures like 31B2 and B3 [1,2,3]. However, complications such as femoral neck shortening, screw sliding, and suboptimal functional recovery have been reported, particularly in cases with severe initial deformity [4,5]. Conventional osteosynthesis techniques, which involve multiple cannulated screws or sliding hip screws, aim to stabilize fractures through compression at the fracture site [6]. While effective for promoting healing, these methods can result in excessive compression during weight-bearing, leading to femoral neck shortening, screw protrusion, and the need for hardware removal, ultimately impacting functional outcomes and quality of life.

Long-threaded screws have shown potential in reducing femoral neck shortening by stabilizing both the proximal and distal trabeculae through threads that cross the fracture line [7,8,9]. However, previous studies have largely focused on radiological and functional outcomes, with limited discussion on optimal insertion positions or techniques. Felton et al. demonstrated in the FAITH trial that femoral neck shortening is significantly associated with inferior hip function, underscoring the clinical importance of minimizing this complication [10]. While the use of short-threaded screws allows for effective fracture site compression, it may lead to complications such as femoral neck shortening, particularly during the weight-bearing phase. Conversely, relying solely on long-threaded screws, which cross the fracture line to stabilize both fragments, may fail to achieve adequate fracture site compression, potentially compromising the healing process. These challenges highlight the necessity of a balanced fixation strategy that incorporates the advantages of both screw types to minimize complications while ensuring stable fracture healing.

Short-threaded screws allow for effective fracture site compression, which is critical for osteosynthesis, particularly in cancellous bone. However, complications such as femoral neck shortening and screw sliding can arise, particularly during the weight-bearing phase. Conversely, fully threaded screws, which cross the fracture line to stabilize both fragments, may reduce sliding and shortening but often fail to achieve adequate initial fracture site compression. In contrast, the sequential fixation technique combines the strengths of both approaches. By initially using short-threaded screws for effective fracture site compression and then replacing them with long-threaded screws to stabilize both proximal and distal fragments, this method addresses the limitations of existing methods. It ensures controlled compression while minimizing complications such as screw sliding and femoral neck shortening.

In this study, the term “long-threaded screws” refers to any screws with threads crossing the fracture line, regardless of whether they are partially threaded (e.g., 32 mm threads) or fully threaded. This definition emphasizes the role of screw threads in engaging both the proximal and distal bone fragments, providing enhanced stability across the fracture line.

We hypothesized that the use of a sequential screw technique, where short-threaded screws are initially used to compress the fracture site followed by replacement with long-threaded screws, could effectively maintain the compressed state and reduce screw sliding, ultimately minimizing femoral neck shortening. This approach aims to stabilize both the proximal and distal fragments while preserving the mechanical advantage of initial fracture compression. The purpose of this prospective observational study was to compare the clinical and radiological outcomes of this sequential screw technique with those of conventional short-threaded screws in the treatment of valgus-impacted femoral neck fractures.

## 2. Materials and Methods

### 2.1. Patient Group

Patients aged 60 years or older with valgus-impacted femoral neck fractures (classified as OTA 31B1.1 or B1.2) treated at our institution between March 2017 and February 2021 were eligible for inclusion in this study. All fractures were managed using a sequential fixation technique, which involved the initial use of short-threaded screws followed by replacement with long-threaded screws. Radiological confirmation of the fracture, along with sufficient preoperative and follow-up imaging, was required. Additionally, only patients with a minimum follow-up period of 12 months were included to ensure comprehensive clinical and radiological assessments.

Patients were excluded if they met any of the following criteria:

1. Pathological fractures, including those caused by malignancy or insufficiency fractures due to severe osteoporosis.
2. Displaced femoral neck fractures (OTA 31B2 or B3) or fractures resulting from high-energy trauma with an Injury Severity Score > 15.
3. Mortality within 12 months postoperatively or insufficient follow-up data, including missing imaging or clinical records.
4. Prior ipsilateral hip surgery or conditions affecting the same hip, such as periprosthetic fractures or severe unrelated deformities.
5. Severe comorbidities that precluded surgery or rehabilitation, including terminal illnesses or conditions impacting ambulation.

This study was conducted prospectively, starting in March 2017. Data were collected in a standardized format, including patient demographics, radiological measurements, surgical details, and follow-up outcomes. The sequential fixation technique became the standard surgical method at our institution in early 2019. To enable a comparative analysis, the study period was extended to include patients treated before its adoption (March 2017–February 2019), who were managed solely with short-threaded screws. Regular follow-ups were conducted postoperatively at six weeks, three months, six months, and one year, and annually thereafter, to gather clinical and radiological data.

### 2.2. Surgical Method and Rehabilitation

Surgery was performed on a fracture table under general or spinal anesthesia with fluoroscopic guidance. Valgus impaction was not disimpacted; however, posterior tilt of the capital fragment (apex anterior angulation) was reduced by internally rotating the leg and applying anterior pressure. Each fracture was initially fixed percutaneously with three 7.0 mm cannulated screws (Synthes, Oberdorf, Switzerland) with a 16 mm thread length, inserted parallel within ~5° without convergence or dispersion. Washers were used when weakening of the lateral femoral cortex was observed.

The three cannulated screws were routinely arranged in an inverted triangle. The infero-central screw was inserted through the lateral cortex of the subtrochanteric area, ensuring it was not distal to the center of the lesser trochanter and was placed close to the medial cortex of the femoral neck. The supero-anterior and supero-posterior screws were then inserted to achieve maximum fixation, forming the three sides of an inverted triangle. Initially, screws with a 16 mm thread length (short-threaded screws) were used, ensuring that the screw threads did not cross the fracture line. Compression at the fracture site was achieved by tightening the screws in the following order: infero-central, supero-anterior, and supero-posterior screws.

After compression, the 16 mm thread length screws were sequentially replaced with screws having a 32 mm thread length or fully threaded screws to stabilize both the proximal and distal fragments. Compression of the infero-central screw facilitated closure of the fracture gap at the thick inferior femoral neck cortex, if present. For patients with fracture lines located at the mid-neck or with a large proximal femur geometry, fully threaded screws were used because 32 mm screws did not adequately engage the distal trabeculae.

Rehabilitation began on the first postoperative day with sitting and continuous passive motion of the knee and hip joints. Standing and ambulation with walking aids were typically allowed within three days post-surgery.

### 2.3. Evaluation

The fracture types were classified according to the AO/OTA system [11]. All radiological assessments and measurements were conducted based on consensus between two evaluators (the first author and the corresponding author). Thirty-one patients had a history of hip fracture on the contralateral side. For these patients, bone mineral density (BMD) was measured at the lumbar spine using dual-energy X-ray absorptiometry (Discovery™, Hologic^®^, Bedford, MA, USA), and results were reported as T-scores to provide a standardized metric for osteoporosis evaluation. For the remaining patients, BMD of the unaffected hip was measured postoperatively, and T-scores of the femoral neck were used to represent their BMD. Body mass index (BMI, kg/m^2^) was calculated at the time of admission, and the American Society of Anesthesiologists (ASA) classification of physical status was determined preoperatively. Clinical outcomes were assessed one year postoperatively by orthopedic residents blinded to patient information using the Harris Hip Score (HHS).

### 2.4. Radiological Measurement

Standard anteroposterior (AP) radiographs of the hip were obtained with both legs positioned at an internal rotation of 15°. Lateral radiographs were taken with the opposite hip flexed and abducted. Angulation at the fracture site was determined from the AP and lateral radiographs by measuring the angulation of the principal trabecular system in the capital fragment, as described for the measurement of the Garden alignment index [12]. The degree of posterior tilt was measured using the method described by Palm et al. [13]. Measurements of posterior and valgus tilt were independently performed twice by the first author and the corresponding author. The average of these four measurements was used to minimize variability and enhance measurement reliability. According to the institution’s protocol, serial radiographs were obtained preoperatively, immediately after surgery, and six weeks, three months, six months, and one year postoperatively, and then at one-year intervals thereafter, if necessary.

For measuring the sliding distance of the screw (SDS), we used the modified method described by Boraiah et al. [14] for the most inferior screw. SDS was measured by two observers (1st and corresponding author) using radiographs that were taken one year after the operation. The mean values thereof were used for analysis.

### 2.5. Data Analysis

Univariate and multivariate analyses were conducted to assess the relationships between demographic and clinical characteristics and outcomes, including SDS, treatment failure, and fixation failure. For continuous outcomes such as SDS, stepwise multiple linear regression with backward elimination was performed to identify independent predictors, using a removal criterion of *p*-value > 0.10. For binary outcomes, such as treatment failure and fixation failure, univariate logistic regression analyses were initially conducted. Variables showing statistical significance in univariate analyses were subsequently entered into multivariate logistic regression models using the forward stepwise likelihood ratio (LR) method to identify independent predictors. All statistical analyses were performed using SPSS software version 29 (IBM Corp., Armonk, NY, USA), with a significance threshold set at *p*-value < 0.05 for all tests.

## 3. Results

**Figure 1:**
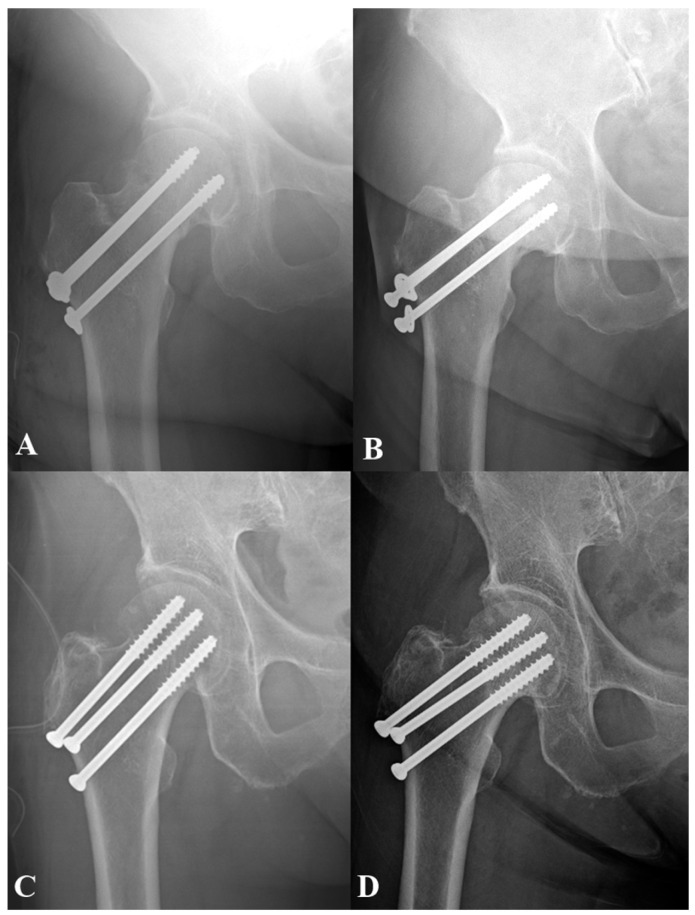
Immediate postoperative X-ray of an 84-year-old woman with a 31B1.2 fracture treated with the conventional short-threaded screw technique (**A**) and the corresponding twelve-month follow-up X-ray (**B**), showing significant screw sliding and femoral neck shortening with avascular necrosis of the femoral head. Immediate postoperative X-ray of an 86-year-old woman with a 31B1.2 fracture treated using the long-threaded screw technique (**C**) and the corresponding twelve-month follow-up X-ray (**D**), demonstrating no evidence of femoral neck shortening or screw sliding.

The demographic and clinical characteristics of the patient group are summarized in Table 1 and Figure 1. The mean age was 75.1 years (range, 61–95 years), with a higher proportion of females (*n* = 108, 80.0%). The mean BMI was 21.8 kg/m^2^ (range, 13.55–32.05 kg/m^2^), and the mean T-score was −2.5 (range, −4.3 to −0.2), reflecting a population with a high prevalence of osteopenia and osteoporosis. The average follow-up period was 38.3 months (range, 12–103 months) (Figure 1).

The analysis of screw sliding distance (SDS) revealed that posterior tilt > 15° was an independent predictor of increased SDS (β = 1.991, standard error = 0.853, *p* = 0.024), as shown in Table 2. Screw type also significantly affected SDS, with the long thread group demonstrating reduced sliding compared to the short thread group (*p* = 0.002). For further details, refer to Table 2.

Table 3 summarizes factors associated with overall failure in treatment, which occurred in 27 cases (20.0%). In the multivariate analysis, posterior tilt > 15° was identified as an independent predictor of treatment failure (OR 5.714, 95% CI: 2.303–14.176, *p* < 0.001). The long thread group was associated with a lower likelihood of failure compared to the short thread group (*p* = 0.030). Detailed results are available in Table 3.

Among the 135 patients included in this study, fixation failure occurred in 14 cases (10.4%), with 11 cases (15.9%) in the short-threaded screw group and 3 cases (4.5%) in the long-threaded screw group, as detailed in Table 1. Fixation failure, a subset of treatment failure, was analyzed in Table 4. Multivariate analysis revealed posterior tilt > 15° (OR 15.085, 95% CI: 2.624–86.716, *p* = 0.002), valgus tilt > 15° (OR 28.616, 95% CI: 3.384–241.955, *p* = 0.002), BMD (OR 0.285, 95% CI: 0.118–0.689, *p* = 0.005), and screw type (long thread; OR 0.062, 95% CI: 0.009–0.431, *p* = 0.005) as significant predictors. For additional findings, refer to Table 4.

## 4. Discussion

In a previous study [4], we confirmed that all valgus-impacted femoral neck fractures healed after osteosynthesis using short-threaded cannulated screws, likely due to the inherent stability of these fractures. However, varying levels of screw sliding and functional deterioration were observed, particularly in cases with severe valgus deformity. These findings highlighted the need for a fixation technique that could stabilize both proximal and distal femoral fragments while minimizing complications such as screw sliding and femoral neck shortening.

Posterior tilt, as evidenced in our results, is a particularly critical factor influencing outcomes. As shown in Table 3, posterior tilt > 15° was a significant predictor of overall complications, and Table 4 further underscores its association with fixation failure (OR 15.085, *p* = 0.002). This finding aligns with our prior research, which demonstrated that greater posterior tilt is strongly associated with inferior clinical outcomes, likely due to its biomechanical disadvantage in fracture stability. Additionally, valgus tilt > 15° was identified as another important predictor of fixation failure (OR 28.616, *p* = 0.002), further emphasizing the need for careful preoperative assessment of both sagittal and coronal alignment.

Importantly, the use of long-threaded screws was shown to significantly reduce the risk of fixation failure (OR 0.062, *p* = 0.005), as illustrated in Table 4. This demonstrates the clinical benefit of transitioning from short-threaded to long-threaded screws, as this approach not only stabilizes both proximal and distal fragments but also mitigates the biomechanical disadvantages posed by severe tilt deformities. Together, these findings underscore the necessity of addressing sagittal and coronal deformities while utilizing an optimized fixation technique to improve outcomes in valgus-impacted femoral neck fractures.

Fracture site compression by the lag screw technique enhances stability and promotes fracture healing in cancellous as well as cortical bone. To achieve effective compression in cancellous bone, all screw threads must pass through the fracture site and anchor in the distal fragment [6,15]. In this configuration, fracture site compression is generated through screw tightening and weight bearing. Frandsen et al. [16] reported that intraoperative compression at the end of sliding hip screw osteosynthesis did not improve outcomes in displaced femoral neck fractures, suggesting that controlled, progressive compression may be more effective.

Finite Element Analysis (FEA) studies have demonstrated that fully threaded screws (FTSs) provide superior biomechanical stability by reducing stress concentrations, fracture displacement, and cancellous bone yielding, particularly when screw threads cross the fracture line to stabilize both proximal and distal fragments [7]. FEA results also suggest that hybrid configurations combining partially threaded screws (PTSs) and FTSs can distribute loads more effectively and prevent varus collapse, further emphasizing the need for techniques that balance compression and stability. These findings provide critical biomechanical support for our approach, which sequentially applies PTSs and FTSs to optimize fixation outcomes.

Femoral neck shortening after osteosynthesis is a common occurrence and exerts a significant negative impact on physical functioning when shortening becomes excessive after bone union [17,18,19,20]. On the other hand, total blocking of screw sliding by inserting a few fully threaded large screws in an X-pattern or locking plate fixation has shown a high failure rate [21,22]. This underscores the need for a new fixation technique that allows controlled sliding during fracture healing. Boraiah et al. [14] attempted similar approaches, using length-stable implants such as three or four fully threaded cannulated screws or sliding hip screws combined with a fully threaded screw. They achieved high union rates with minimal femoral neck shortening in Garden I–IV femoral neck fractures. Our study focused exclusively on valgus-impacted fractures because these fractures are generally treated with osteosynthesis across all age groups.

Parker and Ali [23] performed a randomized clinical trial using short-threaded (16 mm) versus long-threaded (32 mm) screws in 432 intracapsular hip fractures. They concluded that there was no significant difference in fracture healing complications related to screw thread length. However, their study did not assess whether screw threads crossed the fracture line to anchor the distal fragment effectively. In our prospective observational study, we ensured that screw threads crossed the fracture site and stabilized the distal fragment. If one or two screw threads of the 32 mm screw failed to anchor the distal fragment, we switched to fully threaded screws to provide definitive stabilization. To mitigate potential challenges during screw replacement, we adopted a sequential replacement technique. Guide pins were maintained in place throughout the procedure, ensuring that the trajectory of the screws remained consistent. Short-threaded screws were initially inserted over these guide pins to achieve fracture site compression. Subsequently, these screws were replaced sequentially with long-threaded screws, following the same guide pin trajectory. This method minimized the risk of screw misalignment, instability, or deviation during insertion. Additionally, although femoral head perforation is a theoretical concern with long-threaded screws, no such complications were observed in our study or in similar studies utilizing long-threaded or fully threaded screws. This highlights the safety and reliability of the technique when combined with careful surgical execution and fluoroscopic guidance.

**Figure 2:**
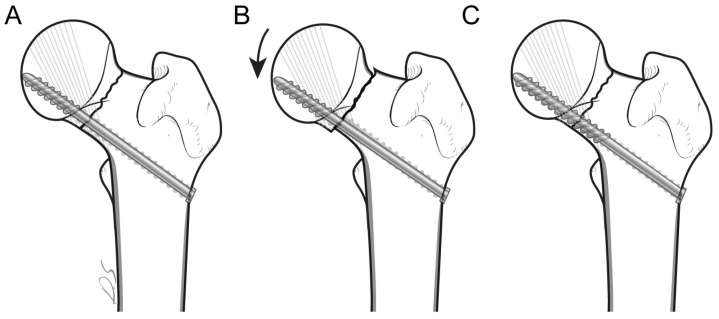
Mechanism of inferior-center screws in femoral neck fractures; (**A**) conventional short-threaded screw shank is not in contact with medial cortex; (**B**) failure mechanism of conventional short-threaded screw after weight bearing in osteoporotic bone; (**C**) long-threaded screw purchases principal compression trabeculae in both fragments and is in contact with the medial femoral neck cortex.

Fixation of osteoporotic cancellous bone using long or fully threaded screws has several mechanical advantages. As illustrated in Figure 2A, conventional cannulated fixation techniques often position the inferior-center screw close to the medial femoral neck cortex to withstand load during weight bearing. However, as the screw threads advance, the screw shank can no longer contact the medial cortex due to its smaller diameter. This mechanical limitation increases the working distance, allowing the femoral head fragment to displace inferiorly during weight bearing until the screw shank recontacts the medial cortex (Figure 2B). This movement weakens distal fixation and damages the supporting trabeculae. In contrast, as shown in Figure 2C, long-threaded screws cross the fracture line and stabilize both fragments by engaging the principal compression trabeculae in both the proximal and distal fragments. This shortens the working distance and ensures continuous contact with the medial femoral neck cortex, minimizing sliding and improving fixation stability during weight bearing.

Furthermore Weil et al. [9] and Schaefer et al. [24] concluded that fully threaded screws significantly improve anteroposterior stiffness and reduce fracture collapse, findings that align with our results. These biomechanical advantages support the efficacy of long-threaded screws in providing stable fixation, reducing femoral neck shortening, and promoting better overall outcomes in fracture management. Cuellar et al. [8] emphasized the biomechanical advantages of hybrid configurations, combining partially threaded and fully threaded screws, to reduce micromotion and enhance stability across the fracture line. Our study aligns with these findings but advances them by using a sequential technique: initial fracture compression with short-threaded screws, followed by replacement with long-threaded screws. This approach ensures comprehensive stabilization without compromising the fracture compression achieved during the initial fixation. Felton et al. [10] underscored the importance of minimizing femoral neck shortening due to its significant impact on functional outcomes. Our results extend this understanding by showing that the sequential fixation technique significantly reduces femoral neck shortening. This reduction indirectly improves functional and radiological outcomes, contributing to better overall recovery.

Concerns exist regarding the use of long-threaded screws, particularly the potential inability to follow the same thread trail created by short-threaded screws after initial compression. Additionally, there is a theoretical risk of screw threads cutting out the femoral head due to increased load concentration. However, in our study, we did not observe any fixation failure or nonunion in valgus-impacted fractures, likely due to their inherent stability. Moreover, the purchasing power of screw threads was found to be greater in the femoral head side, where the trabeculae are denser and better matched to the screw threads, compared to the distal fragment.

This study has several limitations. First, it was not randomized, which limits the ability to establish causality between the fixation techniques and clinical outcomes. Second, although there were no significant differences in demographic and baseline characteristics between the short-threaded and long-threaded screw groups, the retrospective design introduces a potential risk of selection bias. Third, while the follow-up period included some patients with extended follow-up, many cases were concluded at 12 months, making it challenging to evaluate long-term outcomes such as late hardware failure or post-traumatic osteoarthritis. Lastly, as a single-center study, the findings may not be generalizable to broader populations or diverse clinical settings.

## 5. Conclusions

In conclusion, the sequential use of partially threaded screws for initial fracture compression, followed by long-threaded screws for stability, represents a promising technique for managing valgus-impacted femoral neck fractures. This approach effectively balances the need for compression and stability, reducing complications such as screw sliding and femoral neck shortening while improving clinical outcomes.

## Figures and Tables

**Table 1:** Details of cases (*n* = 135).

		Short Screw (*n* = 69)	Long Screw (*n* = 66)	*p*-Value
Sex	Female	57 (82.6%)	51 (47.2%)	0.438
	Male	12 (17.4%)	15 (22.7%)	
Age (years)		75.3 ± 7.7 (62–94)	75.0 ± 8.9 (61–95)	0.412
BMI (kg/m^2^)		22.26 ± 3.11 (17.48–32.05)	21.32 ± 3.13 (13.55–29.09)	0.082
BMD		−2.52 ± 0.99 (−4.30–−0.20)	−2.44 ± 1.14 (−4.30–0.30)	0.678
ASA class	1	20 (29.0%)	15 (22.7%)	0.608
	2	37 (53.6%)	36 (54.5%)	
	3	12 (17.4%)	15 (22.7%)	
Hip Fracture History		17 (24.6%)	14 (21.2%)	0.636
Valgus tilt (>15°)	<15°	36 (52.2%)	30 (45.5%)	0.435
	>15°	33 (47.8%)	36 (57.1%)	
Posterior tilt (>15°)	<15°	46 (66.7%)	43 (65.2%)	0.853
	>15°	23 (33.3%)	23 (34.8%)	
AO/OTA classification	31B1.1	47 (68.1%)	43 (65.2%)	0.715
	31B1.2	22 (31.9%)	23 (34.8%)	
Screw sliding (mm)		3.26 ± 4.01 (0.26–22.34)	1.48 ± 2.33 (0.01–18.23)	0.002
Failure in Treatment	Total	17 (24.6%)	10 (15.2%)	0.168
	AVN	7 (10.1%)	6 (9.1%)	0.836
	Nonunion	6 (8.7%)	5 (7.6%)	0.334
	Fixation failure	11 (15.9%)	3 (4.5%)	0.030
Follow-up (Months)		36.6 ± 21.2 (12–103)	40.2 ± 19.7 (12–92)	0.305
Harris Hip Score		85.5 ± 10.9 (45–100)	87.6 ± 8.1 (60–97)	0.202

BMD, bone mineral density; BMI, body mass index; ASA, American Society of Anesthesiologists; AVN, avascular necrosis of femoral head. Hip Fracture History: hip fracture history of contra-lateral side. Continuous variables were analyzed using the Student’s *t*-test and are presented as mean ± standard deviation (range). Categorical variables were compared using the chi-square test and are expressed as frequencies and percentages.

**Table 2:** Univariate and multivariate linear regression analyses between screw sliding distance and variables.

	Univariate Analysis		Multivariate Analysis	
	Beta ± Standard Error	*p*-Value	Beta ± Standard Error	*p*-Value
Sex (male)	−0.496 ± 0.733	0.500		
Age (years)	0.091 ± 0.034	0.009		
BMI	0.176 ± 0.093	0.060		
BMD	−0.774 ± 0.269	0.005	−0.680 ± 0.218	0.002
Valgus tilt > 15°	2.140 ± 0.558	<0.001	2.090 ± 0.463	<0.001
Posterior tilt > 15°	3.047 ± 0.561	<0.001	2.944 ± 0.487	<0.001
Hip Fracture History	−1.128 ± 0.692	0.105		
Long screw	−1.773 ± 0.567	0.002	−1.906 ± 0.463	<0.001

BMD, bone mineral density; BMI, body mass index. Multivariate linear regression analysis was performed using the stepwise method. At step 4, the model achieved an adjusted R-squared value of 0.379.

**Table 3:** Univariate and multivariate linear regression analyses between complications and variables.

Univariate Logistic Regression Analysis						
Variable	B	S.E.	Wald	*p*-Value	Odds Ratio	95% CI
Sex (female)	1.147	0.478	5.754	0.016	3.149	1.233–8.038
Age (years)	−0.015	0.026	0.327	0.567	0.985	0.935–1.037
BMI (kg/m^2^)	0.074	0.067	1.236	0.266	1.077	0.945–1.228
BMD	−0.430	0.232	3.434	0.064	0.650	0.413–1.025
ASA (ASA 1 vs. ASA 2)	0.223	0.534	0.175	0.676	1.250	0.439–3.559
ASA (ASA 1 vs. ASA 3)	0.323	0.645	0.251	0.617	1.381	0.390–4.884
Hip Fracture History	0.651	0.586	1.235	0.267	1.917	0.608–6.039
Valgus tilt (>15°)	0.804	0.451	3.174	0.075	2.235	0.923–5.415
Posterior tilt (>15°)	1.743	0.464	14.138	<0.001	5.714	2.303–14.176
Screw type (Long)	−0.605	0.443	1.867	0.172	0.545	0.229–1.301
Multivariate logistic regression analysis (Nagelkerke R^2^: 0.169)						
Posterior tilt (>15°)	1.743	0.464	14.138	<0.001	5.714	2.303–14.176

BMD, bone mineral density; BMI, body mass index; ASA, American Society of Anesthesiologists. Multivariate logistic regression analysis was performed using the forward likelihood ratio (LR) method.

**Table 4:** Univariate and multivariate linear regression analyses between fixation failure and variables.

Univariate Logistic Regression Analysis						
Variable	B	S.E.	Wald	*p*-Value	Odds Ratio	95% CI
Sex (female)	−0.916	0.606	2.290	0.130	0.400	0.122–1.311
Age (years)	0.066	0.033	3.950	0.047	1.068	1.001–1.140
BMI (kg/m^2^)	0.152	0.084	3.292	0.070	0.988	1.373
BMD	−1.061	0.406	6.814	0.009	0.346	0.156–0.768
ASA (ASA 1 vs. ASA 2)	0.086	0.640	0.018	0.893	1.090	0.311–3.817
ASA (ASA 1 vs. ASA 3)	−1.210	1.149	1.109	0.292	0.298	0.031–2.835
Hip Fracture History	−0.637	0.793	0.646	0.422	0.529	0.112–2.501
Valgus tilt (>15°)	1.908	0.785	5.903	0.015	6.737	1.446–31.391
Posterior tilt (>15°)	1.775	0.624	8.092	0.004	5.903	1.737–20.059
Screw type (Long)	−1.382	0.676	4.176	0.041	0.251	0.067–0.945
Multivariate logistic regression analysis (Nagelkerke R^2^: 0.503)						
BMD	−1.256	0.451	7.759	0.005	0.285	0.118–0.689
Valgus tilt (>15°)	3.354	1.089	9.482	0.002	28.616	3.384–241.955
Posterior tilt (>15°)	2.714	0.892	9.249	0.002	15.085	2.624–86−716
Screw type (Long)	−2.784	0.991	7.894	0.005	0.062	0.009–0.431

BMD, bone mineral density; BMI, body mass index; ASA, American Society of Anesthesiologists. Multivariate logistic regression analysis was performed using the forward likelihood ratio (LR) method.

## Data Availability

The data that support the findings of this study are available on request from the corresponding author. Due to privacy/ethical restrictions, the data are not publicly available.
